# Virtual Adaptation of an International Exchange Program in Medical Education

**DOI:** 10.5334/aogh.3663

**Published:** 2022-07-08

**Authors:** Celia Fung, Nicholas Maxwell, Stephen Powell, Michelle Benassai, Natalia Chunga, Jennifer Corcoran, William Barbosa, Marisabel Lopez, Betsy Hanampa, Melissa Llaiqui-Condori, Victor Delgado-Lazo, Karina Mendoza, Yanet Astete, Martin Flor, Sheyla Palacios, Blanca Valdovinos, Jorge Risco, Isabel Camargo, Ralph Jozefowicz, Karlo J. Lizárraga

**Affiliations:** 1 Department of Neurology, University of Rochester Medical Center, Rochester, New York, USA; 2 Department of Neurology, Yale School of Medicine, New Haven, Connecticut, USA; 3 Department of Emergency Medicine, Washington University School of Medicine, St. Louis, Missouri, USA; 4 Department of Obstetrics, Gynecology and Reproductive Sciences, Temple University, Philadelphia, Pennsylvania, USA; 5 Departamento de Neurociencias, Facultad de Medicina, Universidad Nacional de San Agustín de Arequipa, Arequipa, PE; 6 La Escuelita AQMED, Arequipa, PE; 7 Department of Medicine, New York Medical College, New York, USA; 8 Department of Obstetrics and Gynecology, Hospital III Yanahuara, Essalud, Arequipa, PE; 9 Servicio de Neurología, Hospital Regional Honorio Delgado Espinoza, Arequipa, PE

**Keywords:** virtual education, medical education, international exchange program, global health, COVID-19

## Abstract

Medical education has drastically transformed during the COVID-19 pandemic. Measures such as adopting telemedicine visits, minimizing the number of trainees on service, discontinuing external rotations, and converting in-person to online didactics have been broadly and swiftly implemented. While these innovations have promoted greater interconnectivity amongst institutions and made continuing medical education possible, international exchange programs in medical education are still largely disrupted. In response to the changing guidelines and restrictions necessitated by the COVID-19 pandemic, the authors used Kern’s six-step approach to design and implement a virtual curriculum to replace the in-person activities of the 2020–2021 Neurology Peru-Rochester exchange program (NeuroPro). Twenty-seven trainees participated in this virtual adaptation. The average daily attendance was ≥85% and the program was rated 9/10 on average in a feedback survey (63% response rate). The median percentage of correct answers during the pre-test was 64% and it increased to 79% during the post-test (P = 0.003). Virtual adaptation of international exchange programs in medical education is feasible to safely continue international collaborative efforts to promote symbiotic building of local expertise and cross-cultural exchange during the ongoing COVID-19 pandemic and beyond.

Neurological disorders are the leading cause of disability worldwide. While nearly 80% of the neurological disease burden comes from low- and middle-income countries, high-income countries have about 70 times the neurological workforce as low- and middle-income countries [1]. With the increasing neurological disease burden in settings with a paucity of neurologists, building local expertise through specialized neurological training worldwide is an indispensable part of sustainable global neurology interventions [2].

The Neurology Peru-Rochester exchange program (NeuroPro) is an international exchange program established in 2019–2020 by institutional agreements between the University of Rochester School of Medicine and Dentistry (URSMD), Rochester, New York, the Universidad Nacional de San Agustin de Arequipa (UNSA), Arequipa, Peru, and the Universidad Nacional Mayor de San Marcos (UNMSM), Lima, Peru. The goals of the NeuroPro are to promote global equity in medical education, foster cross-cultural exchange and cultural humility, and engage in the symbiotic building of local expertise through cultural immersion and educational innovation. The NeuroPro was originally designed to be an annual two-week to four-week, in-person, international elective consisting of clinical and teaching components. The program is designed for 4–6 selected trainees and two faculty members from the URSMD to travel to Peru for two-week rotations [3], and 2–4 selected trainees from the UNSA and the UNMSM to travel to Rochester for four-week rotations each year. Trainee application and selection is carried out by each institution as guided by the respective institutional agreements. Selection is competitive based on personal statements, curricula vitae, transcripts, letters of recommendation, cross-cultural competence, English and Spanish language skills. All eligible trainees are encouraged to apply.

When the COVID-19 pandemic began, institutions across the world had to reimagine training in order to continue medical education while minimizing risks. In response to the changing guidelines and restrictions necessitated by the pandemic, we used Kern’s six-step approach to design and implement a virtual curriculum to replace the 2020–2021 in-person activities of the NeuroPro: 1) problem identification and general needs assessment, 2) targeted needs assessment, 3) goals and measurable objectives, 4) educational strategies, 5) implementation, 6) evaluation and feedback [4].

Participating institutions selected four last-year medical students and three neurology residents from the URSMD, eight last-year medical students and two neurology residents from the UNSA and 10 last-year medical students from the UNMSM. We obtained approval from the institutional review board of the UNSA.

## Problem identification and general needs assessment

Adopting video visits, minimizing the number of trainees on in-person service, and converting in-person to online didactics are measures that have been broadly and swiftly implemented to continue medical education during the COVID-19 pandemic. Tele-education is non-inferior to in-person lectures and confers the benefit of allowing synchronous (i.e., simultaneous) and asynchronous exposure to a variety of educators and topics across institutions [5,6]. While these innovations have promoted greater interconnectivity amongst institutions and made continuing medical education possible, international initiatives, electives, and projects continue to be heavily disrupted, if not completely halted. In this context, we chose the widely available video conferencing service Zoom to ensure synchronous video and audio participation of faculty and trainees during this international virtual education effort. Synchronous activities were prioritized anticipating the need for live exchange to overcome potential language barriers.

## Targeted needs assessment

To ensure that the curriculum centered on the interests and needs of learners from participating institutions, planning meetings were held every two weeks after the trainee selection process was finalized. Topics of interest were proposed and selected during open discussions between participating faculty and trainees (Table 1). Some of these topics are already covered by the trainee curricula at each institution. However, it was agreed that the additional cross-cultural training provided as part of this program would be beneficial for present and future participants.

**Table 1:** Topics of interests for the NeuroPro 2020–2021 as selected during the targeted needs assessment step of the Kern’s approach.


NEUROLOGY TOPICS	GENERAL TOPICS

Neurological exam Neuroanatomical localization Case-based discussions of common neurological conditions in the USA Case-based discussions of common neurological conditions in Peru Evidence-based neurology Tele-neurology	Medical English and Spanish focused on neurology Local epidemiology, basic statistics Evidence-based medicine, medical informatics ECG interpretation Cardiopulmonary resuscitation skills Labor and delivery skills Cross-cultural exchange, healthcare systems and global health equity


## Goals and measurable objectives

Additional meetings were held amongst participating faculty and trainees to define specific objectives and facilitate lesson planning for the identified topics of interest (Table 1). To obtain a short-term measure of intervention effect, faculty members prepared 1–2 multiple-choice questions to evaluate specific objectives corresponding to each topic. Questions were designed to be answered in ≤1 minute. We compiled 20 questions to create the pre-test and post-test, which were built in as anonymous polls to be administered via Zoom on the first day and last day of the program, respectively. We prepared an anonymous feedback survey with nine questions using the online program SurveyMonkey. We planned to send English and Spanish versions of this survey to USA-based and Peru-based participants, respectively, on the last day of the program. We planned for participants to have up to 48 hours to complete the survey (Supplementary Material).

## Educational strategies

The virtual curriculum was designed to include four-hour synchronous activities per day over 10 days (two weeks). Based on the topics of interest (Table 1), activities were divided into two-hour general and two-hour neurology segments. Ten-minute breaks were built in every 45–50 minutes. Faculty and trainees were assigned topics based on their expertise and interests. Trainees were paired with faculty for resources and guidance and they were assigned topics according to their level of training. To provide diverse learning experiences, the curriculum was designed to include: 1) lectures, 2) case-based learning, 3) skills workshops including simulations, 4) standardized patient encounters in breakout rooms, 5) live tele-neurology consultations presented by local residents to J. Risco or K. Lizarraga (both licensed to practice medicine in Peru), 6) cross-cultural exchange discussions and 7) journal club (Table 2). Materials and articles were chosen to be shared electronically with trainees and faculty before each activity.

**Table 2:** Virtual educational activities and schedule of the NeuroPro 2020–2021.


	GENERAL SEGMENT	NEUROLOGY SEGMENT

**Week 1**		

Day 1	Lecture: Local epidemiology and basic statistics (M. Llaiqui, faculty, Peru)	Faculty and trainee introductions Cross-cultural exchange 1: Effects of the pandemic in our local practices Pre-Test

Day 2	Lecture: Neurological examination 1 (J. Risco, faculty, USA)	Lecture: Neurological examination 2 (N. Chunga and J. Risco, resident and faculty, USA) Case-based learning: Localization (N. Chunga and J. Risco, resident and faculty, USA)

Day 3	Skills workshop: Local epidemiology, basic statistics (M. Llaiqui, faculty, Peru)	Lecture: Tele-neurology basics (J. Risco and K. Lizarraga, faculty, USA) Case-based learning: Epilepsy (M. Lopez and I. Camargo, resident and faculty, Peru)

Day 4	Lecture: Evidence-based medicine (K. Lizarraga, faculty, USA)	Case-based learning: Ischemic stroke (J. Corcoran and J. Risco, resident and faculty, USA) Case-based learning: Hemorrhagic stroke (W. Barbosa and J. Risco, resident and faculty, USA)

Day 5	Skills workshop: ECG (N. Maxwell and V. Delgado-Lazo, student and resident, USA)	Live tele-neurology consultation 1 (Stroke) (B. Hanampa, Peru, and J. Risco, USA, resident and faculty) Case-based learning: Neuro-infectious disease (I. Camargo, faculty, Peru)

**Week 2**		

Day 6	Lecture: Medical informatics (K. Lizarraga, faculty, USA)	Case-based learning: Neuromuscular (C. Fung and K. Lizarraga, student and faculty, USA) Case-based learning: Headache (S. Powell and K. Lizarraga, student and faculty, USA)

Day 7	Skills workshop: Labor and delivery simulation (M. Benassai, USA, and K. Mendoza, Peru, student and faculty)	Case-based learning: Movement disorders 1 (Parkinson’s disease) (K. Lizarraga, faculty, USA) Live tele-neurology consultation 2 (Parkinson’s disease) (M. Lopez, Peru, and K. Lizarraga, USA, resident and faculty)

Day 8	Skills workshop: Cardiopulmonary resuscitation simulation (N. Maxwell and V. Delgado-Lazo, student and resident, USA)	Case-based learning: Movement disorders 2 (B. Valdovinos, faculty, USA) Case-based learning: Neuro-immunology (B. Hanampa and I. Camargo, resident and faculty, Peru)

Day 9	Skills workshop: Medical English (M. Benassai, W. Barbosa, N. Chunga, B. Valdovinos, student, residents and faculty, USA)	Standardized patient encounters: Medical English (M. Benassai, W. Barbosa, N. Chunga, B. Valdovinos, student, residents and faculty, USA)

Day 10	Lecture: Critical appraisal of the literature (K. Lizarraga, faculty, USA)	Journal club Cross-cultural exchange 2: health care systems, global equity Post-Test Feedback survey


## Implementation

Synchronous virtual activities were held between March 1 and March 12, 2021, from 4–8 p.m. EST (3–7 p.m. Peru) using the platform Zoom. Selected materials and articles were shared electronically with trainees and faculty before each activity on a daily basis. To adapt to the diverse language skills of participants, a mixture of English and Spanish language was allowed for communication. Participants were encouraged to use the “raise hand” and Chat functions in Zoom to ask questions, make comments or share content during the program.

## Evaluation and feedback

Average attendance was 85% for the general segment and 90% for the neurology segment. We used the Wilcoxon signed-rank test to compare the median percentage of correct answers per question during the pre-test and post-test. This percentage increased from 64% during the pre-test (interquartile range 50–74%) to 79% during the post-test (interquartile range 61–86%) (P = 0.003). Seventeen of the 27 trainees completed the anonymous feedback survey (63% response rate). Average rating of the program was nine on a 1–10 point scale. The most frequently mentioned positive aspects were the live tele-neurology consultations, cross-cultural discussions surrounding healthcare delivery systems, and the opportunity to network with international faculty and trainees. The most frequently mentioned negative aspects were screen/Zoom fatigue and issues with internet connection.

## Discussion

Virtual international programs in medical education have been piloted in various capacities [7,8]. We followed Kern’s six-step approach to design and implement a virtual curriculum for the NeuroPro 2020–2021 [4]. This virtual modification was feasible and it allowed us to host 27 trainees without incurring in travel or lodging expenses. This two-week program consisted of four-hour synchronous activities per day, which accommodated two-hour general and two-hour neurology segments with breaks (Table 2). This approach allowed coverage of all topics prioritized during the targeted needs assessment (Table 1). However, participants experienced screen/Zoom fatigue. Prior studies have found that trainees prefer a combination of synchronous and asynchronous components. Medical students might prefer to watch recorded lectures in an asynchronous fashion [9]. Furthermore, it has been found that trainees tolerate synchronous virtual activities for approximately three hours per day. We prioritized synchronous activities anticipating the need for live exchange to overcome language barriers. Future virtual programs could reduce the number of hours of synchronous activities and incorporate more asynchronous activities to minimize the risk for screen fatigue [10].

Another limitation we encountered was the intermittent unreliability of internet connection for some participants. This issue was particularly troublesome during the live tele-neurology consultations. Utilizing stable and higher-speed internet connections facilitated by participating institutions could be considered for future programs. Virtual platforms that require less internet bandwidth for synchronous video capabilities could also be considered.

We decided to use case-based learning presentations to cover most of the neurology topics. As previously demonstrated, these activities promoted engagement and facilitated discussions during this virtual program. The addition of skills workshops, simulations and standardized patient encounters in breakout rooms was feasible and provided the opportunity for live demonstrations that increased participant engagement. The concomitant use of the “raise hand” and Chat functions in Zoom also increased active participation during the program.

The idea of universal adoption of tele-neurology has been recently embraced due to pandemic-related policy changes. Thus, we deliberately included live tele-neurology consultations to equip students with skills unique to tele-neurology visits. These activities were considered a strength by most participants as they provided real-world opportunities to consolidate knowledge while contrasting social determinants of health and health care delivery systems. While it is unclear the exact role that tele-neurology will play beyond the pandemic, it has been projected that there is likely to be a more permanent transition to tele-health. The ever-expanding digital infrastructure could allow this transition to be feasible for the growing field of global health [11].

During the ongoing COVID-19 pandemic and beyond, international exchange programs in medical education could be adapted and implemented in a virtual fashion. Virtual modification of these programs is feasible to safely continue international collaborative efforts to promote symbiotic building of local expertise and cross-cultural exchange in medical education.

## Additional Files

The additional files for this article can be found as follows:

10.5334/aogh.3663.s1 Supplementary Material 1. Multiple choice questions used during the pre-test and post-test of the NeuroPro 2020–2021.

10.5334/aogh.3663.s2 Supplementary Material 2. Questions of the feedback survey used to evaluate the NeuroPro 2020–2021.
