# Supplementary material for: Virtual Adaptation of an International Exchange Program in Medical Education
Source: Ann Glob Health. 2022 Jul 8;88(1):52. doi: 10.5334/aogh.3663 (PMC9267016; doi:10.5334/aogh.3663)
Supplement: Supplementary Material 1. — Multiple choice questions used during the pre-test and post-test of the NeuroPro 2020–2021. [file agh-88-1-3663-s1.pdf]

Supplementary Material 1.

Multiple choice questions used during the pre-test and post-test of the NeuroPro 2020–2021.

Question 1

Which of the following clinical signs is consistent with upper motor neuron dysfunction?

A. Rigidity, pronator drift, fasciculations

B. Flaccidity, atrophy, Babinski sign

C. Spasticity, pronator drift, hyporeflexia

D. Hyperreflexia, pronator drift, Babinski *(correct answer)*

Question 2

A positive Romberg test indicates dysfunction of the…

A. Cerebellar vermis

B. Proprioceptive system *(correct answer)*

C. Visual system

D. Basal ganglia

Question 3

A 65-year-old man has difficulties using silverware during breakfast. His wife states that he correctly asked for a spoon but tried to use it as a knife. His exam did not reveal numbness or incoordination. Which of the following describes this presentation?

A. Aphasia

B. Apraxia *(correct answer)*

C. Ataxia

D. Anosognosia

Question 4

In which of the following subspecialties of neurology has it not been demonstrated that telemedicine improves diagnostic accuracy?

A. Neuromuscular *(correct answer)*

B. Dementia

C. Headache

D. Multiple sclerosis

Question 5

Which of the following parts of the neurological examination cannot be reliably performed and interpreted over a video visit?

A. Mental status

B. Fundoscopy *(correct answer)*

C. Reflexes

D. Gait

Question 6

For the last 6 months, a 20-year-old woman has had episodes of epigastric pain that ascend to her throat, followed by anxiety and loss of awareness for 1–2 minutes. Neurological exam, EEG and MRI brain are normal. Which of the following is the next best step?

A. Treat gastroesophageal reflux

B. Treat anxiety

C. Repeat EEG to capture episodes

D. Start antiepileptic therapy *(correct answer)*

Question 7

Which of the following types of epileptic seizures is more frequent in patients with a brain abscess?

A. Generalized tonic-clonic

B. Generalized absence

C. Focal onset *(correct answer)*

D. Unknown onset

Question 8

A 65-year-old man presents with left sided weakness for the last 2 days that became much worse 2 hours ago. His blood pressure is 130/60 mmHg. He has dysarthria, left pronator drift and sensory extinction. Which of the following contraindicates using tPA?

A. Time of onset *(correct answer)*

B. Age

C. Blood pressure

D. Unknown glycemia

Question 9

A 67-year-old woman complains of sudden onset of a very severe, global headache and nausea that she had not experienced before. Her headache resolved. Her neurological exam and computed tomography of the brain are normal. What is the next best step?

A. Echocardiogram

B. Aspirin

C. Lumbar puncture *(correct answer)*

D. Follow-up in clinic

Question 10

A 50-year-old man presents with headache and fever. Exam reveals meningeal signs. CSF is crystal clear with 25 cells/mm^3 (60% mononuclear), 35 mg% of protein and 55 mg% of glucose. Which one is the most likely diagnosis?

A. Bacterial meningoencephalitis

B. Viral encephalitis *(correct answer)*

C. Meningoencephalitis due to TB

D. Fungal meningoencephalitis

Question 11

Which one is true regarding viral encephalitis?

A. Patients present with fever and altered mental status *(correct answer)*

B. EEG is not specific

C. Herpes encephalitis tends to affect the parietal lobes

D. CSF is purulent

Question 12

What is the molecular mechanism that predominantly drives Guillain-Barré syndrome?

A. B-cell mediated molecular mimicry *(correct answer)*

B. Abnormal amyloid accumulation

C. Cytotoxic T-cell mediated inflammation

D. Bacterial or viral attack on myelin protein

Question 13

What is the classic CSF finding in a patient with acute inflammatory demyelinating polyradiculoneuropathy?

A. Elevated white cell count with neutrophilic predominance

B. Albumino-cytologic dissociation *(correct answer)*

C. Markedly decreased glucose with elevated protein

D. Elevated red cell count with xanthochromia

Question 14

Which of the following is a “red flag” in a patient who presents with headache?

A. The patient is pregnant *(correct answer)*

B. The headache is similar to prior episodes

C. There are no additional neurological symptoms

D. Complete neurological examination is normal

Question 15

Which of the following is not a trigeminal autonomic cephalalgia?

A. Cluster headache

B. Paroxysmal hemicranias

C. Trigeminal neuralgia *(correct answer)*

D. Short-lasting neuralgiform headache attacks with conjunctival injection and tearing

Question 16

Which of the following types of tremor can be treated with deep brain stimulation of the ventral intermediate nucleus of the thalamus?

A. Enhanced physiologic tremor

B. Essential tremor *(correct answer)*

C. Functional tremor

D. Drug-induced tremor

Question 17

Which of following are the cardinal motor features of Parkinson's disease?

A. Resting tremor, rigidity, bradykinesia and postural instability *(correct answer)*

B. Action tremor, rigidity, bradykinesia and postural instability

C. Resting tremor, spasticity, bradykinesia and ataxia

D. Action tremor, spasticity, bradykinesia and ataxia

Question 18

A 60-year-old woman presents with dizziness when she stands up. Her blood pressure sitting is 120/80 mmHg and drops to 90/60 mmHg when standing. On exam, she has symmetric increased tone and slowness of movement. She has no tremor. Her gait is slightly wide-based. What is her most likely diagnosis?

A. Idiopathic Parkinson's disease

B. Multiple Systems Atrophy *(correct answer)*

C. Dementia with Lewy Bodies

D. Progressive Supranuclear Palsy

Question 19

Which of the following conditions can manifest with brain granulomas?

A. Tuberculosis

B. Sarcoidosis

C. Granulomatosis with polyangiitis (Wegener)

D. All of the above *(correct answer)*

Question 20

Which of the following is most useful in the differential diagnosis of cerebral granulomatosis?

A. Anti-NMDA

B. Anti-GAD

C. ANCA *(correct answer)*

D. Anti-Hu
