# Supplementary material for: Virtual Adaptation of an International Exchange Program in Medical Education
Source: Ann Glob Health. 2022 Jul 8;88(1):52. doi: 10.5334/aogh.3663 (PMC9267016; doi:10.5334/aogh.3663)
Supplement: Supplementary Material 2. — Questions of the feedback survey used to evaluate the NeuroPro 2020–2021. [file agh-88-1-3663-s2.pdf]

Supplementary Material 2.

Questions of the feedback survey used to evaluate the NeuroPro 2020–2021.

English version:

1. On a scale from 1–10, how would you rate this course overall?

2. Was this program what you expected? If not, how was it different from what you expected? (Please be as specific as possible)

3. What was your favorite part of this program?

4. What was your least favorite part of this program?

5. Was the timing of activities and the amount of time this program required appropriate? If not, what/how would you like it to be different in the future? (Please be as specific as possible)

6. In your opinion, how was the balance between English and Spanish that was utilized in this program?

7. Would you recommend this program to a friend? Why?

8. What should we do differently in the future? (Please be as specific as possible)

9. Please write here any additional comments you may have. Thank you.

Spanish version:

1. En una escala del 1 al 10, ¿cómo calificaría este programa en general?

2. ¿Este programa fue lo que esperaba? Si no fue así, ¿qué diferencias existieron con respecto a lo que esperaba? (Por favor sé lo más específico que puedas)

3. ¿Cuál fue su parte favorita de este programa?

4. ¿Cuál ha sido la parte que menos le ha gustado de este programa?

5. ¿Fue apropiado el horario de las actividades del programa y la cantidad de tiempo que requirió este programa? Si no fue así, ¿qué te gustaría que fuera diferente en el futuro? (Por favor sé lo más específico que puedas)

6. En tu opinión, ¿qué te pareció el equilibrio entre el idioma inglés y el idioma español que se utilizó en este programa?

7. ¿Recomendarías este programa a un amigo? ¿Por qué?

8. ¿Qué deberíamos hacer de forma diferente en el futuro? (Por favor sé lo más específico que puedas)

9. Por favor escribe aquí cualquier comentario adicional que tengas. Gracias.
